# Effects of mixed oil emulsion on short-term clinical outcomes in premature infants: A prospective, multicenter, randomized controlled trial

**DOI:** 10.1038/s41430-023-01288-6

**Published:** 2023-05-03

**Authors:** Qing Yang, Juan Kong, Rui-Miao Bai, Wen-Ting Yu, Juan Zhang, Wei Shen, Li-Xia Tang, Yao Zhu, Ya-Sen Wang, Si-Yu Song, Dong Yang, Shi-Rong Song, Yi-Jia Zhang, Xin-Zhu Lin, Fan Wu, Zhan-Kui Li, Jian Mao, Xiao-mei Tong

**Affiliations:** 1 Department of Neonatology, Women and Children’s Hospital, School of Medicine, Xiamen University, Xiamen, 361003 China; 2 Xiamen key laboratory of perinatal-neonatal infection, Xiamen, China; 3 Xiamen Clinical Research Center for Perinatal Medicine, Xiamen, China; 4 Department of Neonatology, The Third Affiliated Hospital of Guangzhou Medical University, Guangzhou, 510150 China; 5 Department of Neonatology, Northwest Women’s and Children’s Hospital, Xian, Shanxi 710061 China; 6 Department of Neonatology, Shengjing Hospital of China Medical University, Shenyang, Liaoning 110004 China; 7 Department of Pediatrics, Peking University Third Hospital, Beijing, 100191 China

**Keywords:** Randomized controlled trials, Nutrition disorders

## Abstract

**Objective:**

This study compared the clinical effects of two different lipid emulsions in premature infants with gestational age < 32 weeks (VPI) or birth weight < 1500 g (VLBWI) to provide an evidence-based medicine basis for optimizing intravenous lipid emulsion.

**Methods:**

This was a prospective multicenter randomized controlled study. A total of 465 VPIs or VLBWIs, admitted to the neonatal intensive care unit of five tertiary hospitals in China from March 1, 2021 to December 31, 2021, were recruited. All subjects were randomly allocated into two groups, namely, medium-chain triglycerides/long-chain triglycerides (MCT/LCT) group (*n* = 231) and soybean oil, medium-chain triglycerides, olive oil, and fish oil (SMOF) group (*n* = 234). Clinical features, biochemical indexes, nutrition support therapy, and complications were analyzed and compared between the two groups.

**Results:**

No significant differences were found in perinatal data, hospitalization, parenteral and enteral nutrition support between the two groups (*P* > 0.05). Compared with the MCT/LCT group, the incidence of neonates with a peak value of total bilirubin (TB) > 5 mg/dL (84/231 [36.4% vs. 60/234 [25.6%]), a peak value of direct bilirubin (DB) ≥ 2 mg/dL (26/231 [11.3% vs. 14/234 [6.0%]), a peak value of alkaline phosphatase (ALP) > 900 IU/L (17/231 [7.4% vs. 7/234 [3.0%]), and a peak value of triglycerides (TG) > 3.4 mmol/L (13/231 [5.6% vs. 4/234[1.7%]]) were lower in the SMOF group (*P* < 0.05). Univariate analysis showed that in the subgroup analysis of < 28 weeks, the incidence of parenteral nutrition-associated cholestasis (PNAC) and metabolic bone disease of prematurity (MBDP) were lower in the SMOF group (*P* = 0.043 and 0.029, respectively), whereas no significant differences were present in the incidence of PNAC and MBDP between the two groups at > 28 weeks group (*P* = 0.177 and 0.991, respectively). Multivariate logistic regression analysis revealed that the incidence of PNAC (a*RR*: 0.38, 95% confidence interval [*CI*]: 0.20–0.70, *P* = 0.002) and MBDP (a*RR*: 0.12, 95% CI: 0.19–0.81, *P* = 0.029) in the SMOF group were lower than that in the MCT/LCT group. In addition, no significant differences were recorded in the incidence of patent ductus arteriosus, feeding intolerance, necrotizing enterocolitis (Bell’s stage ≥ 2), late-onset sepsis, bronchopulmonary dysplasia, intraventricular hemorrhage, periventricular leukomalacia, retinopathy of prematurity and extrauterine growth retardation between the two groups (*P* > 0.05).

**Conclusions:**

The application of mixed oil emulsion in VPI or VLBWI can reduce the risk of plasma TB > 5 mg/dL, DB ≥ 2 mg/dL, ALP > 900 IU/L, and TG > 3.4 mmol/L during hospitalization. SMOF has better lipid tolerance, reduces the incidence of PNAC and MBDP, and exerts more benefits in preterm infants with gestational age < 28 weeks.

## Introduction

Very preterm infants (VPI) with gestational age (GA) < 32 weeks or very low birth weight infants (VLBWI) with birth weight (BW) < 1500 g in the neonatal intensive care unit (NICU) are completely or partially dependent on parenteral nutrition (PN) in the early postnatal period to meet the nutrient and energy requirements of growth due to the presence of an immature digestive system. Early active PN is known to improve the survival rate and prognosis of VPI or VLBWI. Intravenous lipid emulsions (ILE) are considered a standard and essential component of PN. ILE ensures proper weight gain in premature infants, provides essential fatty acids and energy, promotes the development of brain nerves and retina, reduces the related complications of premature infants, and improves the success rate of rescue [1]. However, long-term PN can lead to serious complications, such as metabolic disorder, catheter-related bloodstream infection, systemic inflammatory response syndrome, late-onset sepsis (LOS), gastrointestinal mucosal atrophy, and flora imbalance [2], also includes parenteral nutrition-associated cholestasis (PNAC). The incidence of PNAC is between 25% and 60% among children who receive long-term PN as reported [3]. PNAC can develop into liver fibrosis, cirrhosis, liver failure, and even death without prompt treatment.

In the 1960s, Professor Wretlind of Sweden developed a fat emulsion based on soybean oil [4], known as the traditional soybean oil fat emulsion Intralipid. It contains lots of omega-6 long-chain polyunsaturated fatty acids (ω-6 LCPUFAs) and phytosterols, which are important causes of PNAC [5]. Intralipid is presently gradually replaced by medium chain triglycerides/long chain triglycerides (MCT/LCT), olive oil (OO), and soybean oil-medium chain triglycerides-olive oil-fish oil (SMOF). MCT/LCT, composed of 50% MCT and 50% LCT mixed physically, is widely used in China. LCT provides essential fatty acids. MCT can be rapidly metabolized into lipids, with no need for carnitine to transport to mitochondria, which can reduce lipid peroxidation and intrahepatic cholestasis [6]. Therefore, the Guidelines for Clinical Application of Neonatal nutritional support in China (2013) [7] recommend the use of 20% fat emulsion (grade A evidence) in PN support of preterm infants, and MCT/LCT was reported to be better than LCT (Grade B evidence). In 2016, SMOF was approved by the Food and Drug Administration for use in the United States as an equivalent alternative to Intralipid (Baxter Healthcare Corporation, Deerfield, IL) [8]. Recently, SMOF has been used frequently clinically. It is a new fat emulsion composed of 30% soybean oil, 30% MCT, 25% OO, and 15% fish oil and is rich in antioxidant vitamin E (200 mg/L) and α-tocopherol. It has a good balanced fatty acid pattern and ideal ω-6/ω-3 ratio (2.5:1) [6]. SMOF combines the advantages of a variety of oils, and currently, it is considered to be an “ideal” fat emulsion. However, it is still controversial whether SMOF is superior to MCT/LCT. Therefore, we hypothesized that administration of SMOF among VPI and VLBWI could improve the clinical outcomes during hospitalization, especially reducing the incidence of PNAC.

## Materials and Methods

### Study population

This was a prospective, multicenter, randomized controlled study. The subjects were recruited from five tertiary hospitals in China from Mar 1, 2021 to Dec 31, 2021. These five hospitals are composed of Women and Children’s Hospital of Xiamen University, The Third Affiliated Hospital of Guangzhou Medical University, Northwest Women’s and Children’s Hospital, Shengjing Hospital of China Medical University and Peking University Third Hospital, which are located in the north, Northwest, South China, and each hospital has more than 100 NICU beds. The study groups were created according to randomly assigned numbers. All infants who met the standard of inclusive criteria were assigned random numbers and divided into MCT/LCT group or SMOF group. The inclusion criteria were as follows: (1) premature infants with GA < 32 w or BW < 1500 g; (2) estimated duration of PN > 14 days; (3) starting time of PN < 48 h; and (4) premature infants born in or transferred to our hospital within 24 h after birth. The discharge criteria were as follows: (1) the primary disease was cured, and the vital signs were stable; (2) oral feeding and the amount of milk reached 150 mL/kg/d; (3) BW was more than 1800 to 2000 g; and (4) the corrected GA ≥ 36 weeks. Infants with congenital intrauterine infection, congenital malformation, genetic metabolic disease, ABO or Rh hemolytic disease, or who died within 14 days after birth or continuous intravenous ILE ≤ 14 days were excluded.

The study is registered with the Chinese clinical trial registry (http://www.chictr.org.cn/), registration number: ChiCTR210041910. The study was approved by the Ethics Committee of Women and Children’s Hospital, School of Medicine, Xiamen University (2021-01-08, KY-2020–0086–01). The informed consent was obtained from the family members of the children.

### Sample size calculation

The incidence of PNAC is reported to range from 4.27% to 14.07% [9, 10], and the allowable error δ is 2%, α = 0.05, the calculated sample size was 354 cases. We included 400 cases, 200 cases in each group. The average sample size of each hospital was estimated to be 80 cases.

### Data collection

According to the unified questionnaire, the following data were collected: the primary observation index was the incidence of PNAC, and the secondary observation index was blood biochemical indicators: total bilirubin (TB), direct bilirubin (DB), alanine aminotransferase (ALT), aspartate aminotransferase (AST), alkaline phosphatase (ALP), triglyceride (TG), cholesterol (CHOL), serum calcium (Ca), serum phosphorus (P); and early complications related to premature infants were as follows: patent ductus arteriosus (PDA), retinopathy of prematurity (ROP), bronchopulmonary dysplasia (BPD), intraventricular hemorrhage (IVH), periventricular leukomalacia (PVL), feeding intolerance (FI), extrauterine growth retardation (EUGR), LOS, and metabolic bone disease of prematurity (MBDP) were observed. In addition, the general information and perinatal data of the two groups were collected and included GA, BW, gender, 5 min Apgar score, mode of delivery, small for gestational age (SGA), maternal pregnancy complications (gestational hypertension and gestational diabetes mellitus); primary diseases (neonatal respiratory distress syndrome [NRDS] and early-onset sepsis [EOS]) were observed at admission; nutritional status of the two groups during hospitalization: use of amino acids, fat emulsion, PN time, time of starting enteral feeding, time of reaching the whole intestinal feeding, age of reaching the standard of a total caloric, weight growth velocity (GV), days to regain birth weight, maximum physiological weight loss (%), and total hospitalization time (d); and major treatment measures, including mechanical ventilation time (d), non-invasive auxiliary ventilation time (d), and total oxygen consumption time (d).

### Data management and quality control

Before starting the study, all units performed training in strict accordance with the requirements of the research program. Epi Data 3.1 software was used to collect the data and conduct consistency check by two clinic doctors. All participating units collected the data in real-time and uploaded the relevant data after the discharge of children, and locked the database after verification. The technical personnel of the team leader kept close contact with all participating units at any time to check case records, verify the accuracy of data, and solve possible problems in time.

### Nutritional strategies

All enrolled children in the five research centers followed the Guidelines for Clinical Application of Neonatal Nutritional Support in China (2013) [7]. The lipid emulsions used for preterm neonates in NICU were MCT/LCT (20% Lipofundin MCT/LCT, B. Braun Melsungen AG, Germany) and SMOF (20% mixed oil fat injection, Fresenius kabi asutria GmbH, Austria). The composition of the two kinds of fat emulsion is shown in Table 1. The initial dose of lipid emulsion was 1.0 g/kg/d, and the dose was gradually increased to 3.0–3.5 g/kg/d. When TG > 2.26 mmol/L, the fat emulsion was reduced by half when TG > 2.26 mmol/L, and when TG > 3.4 mmol/L, the use of fat emulsion was suspended until clearance. The initial dose of amino acids was 1–2 g/kg/d, and gradually increased to 3.0–4.0 g/kg/d. The glucose infusion rate was started at 4–8 mg/kg/min and was adjusted according to the blood glucose levels; the maximum was no more than 11–14 mg/kg/min. Fat-and water-soluble vitamins were added to the nutrient solution. The feeding protocol was to introduce breast milk soon after birth; breast milk was the first choice. Usually, breast milk/preterm formula was started from trophic feeding 10 mL/kg/day for 3–5 days and subsequently advanced to a feeding amount of 10–20 mL/kg/day. When enteral feeding reached 80 to 100 mL/kg/d, fortification with human milk fortifier (HMF) was initiated. When enteral feeding reached 130–150 mL/kg/d, PN was stopped.

**Table 1:** Main components of two kinds of fat emulsion.

Component	MCT/LCT (C8–C24)	SMOF (C6–C24)
Soybean oil (g/100 mL)	10	6
MCTs (g/100 mL)	10	6
Olive oil (g/100 mL)	-	5
Fish oil (g/100 mL)	-	3
Egg yolk phospholipids (g/100 mL)	1.2	1.2
Glycerol (g/100 mL)	2.5	2.5
α-Tocopherol (mg/L)	11	20
Vitamin E (mg/L)	14	200
Phytosterol (mg/L)	370	207
Energy (kcal/100 mL)	190.8	200
pH	6.5~8.5	7.5~8.8
Osmotic pressure (mOsm/L)	273	265
ω-6: ω-3	7:1	2.5:1

### Study definitions and diagnostic criteria

(1) The definition of SGA: BW of newborns was below the 10^th^ percentile of the average BW of the same-sex infants of the same gestational age; (2) PNAC [11]: when PN > 14 days, skin yellow staining and/or stool color became lighter. At any time, when DB ≥ 34 µmol/L (2 mg/dL) or TB ≤ 85 µmol/L, DBIL ≥ 17 µmol/L (1 mg/dL), and jaundice caused by infectious such as viruses, bacteria, fungi, and malformations of the biliary tract were excluded; (3) PDA [12]: persistence of patent ductus arteriosus > 72 h after birth; hemodynamically significant patent ductus arteriosus (hsPDA): PDA diameter > 1.5 mm, left atrial diameter/aortic diameter ≥ 1.4 or left ventricular end diastolic diameter/aortic diameter ≥ 2.1; accompanied by one of the following clinical manifestations: heart murmur, tachycardia (lasting more than 160 beats/min), increased breathing, increased pulse pressure (> 25 mmHg), hypotension, pulse or cardiac enlargement; (4) ROP: refer to the guidelines for oxygen therapy and prevention and treatment of retinopathy in premature infants (revised edition) [13]; ROP requiring intervention: ROP requiring intravitreal drug injection, laser therapy, or surgery; (5) BPD: newborns with persistent oxygen dependence ≥ 28 days after birth were graded according to the corrected gestational age of 36 weeks or the oxygen demand at discharge: 1) mild: no oxygen use; 2) moderate: with a fraction of inspired oxygen (FiO_2_) < 30%; 3) severe: FiO_2_ ≥ 30% or positive pressure ventilation or mechanical ventilation [14]; (6) Fi: gastric residuals up to 25–50% of the previous feeding, abdominal distension or bloody stools, vomiting or bile reflux after repeated feeding, coffee-like substances in the stomach [15]; (7) EUGR: postmenstrual age of 36 weeks or discharge weight below the 10th percentile of the 2013 Fenton curve for children of the same GA and sex [16]; (8) clinical diagnosis and diagnosis criteria of EOS and LOS, refer to the Expert Consensus on the Diagnosis and Management of Neonatal Sepsis (version 2019) [17]; (9) MBDP: ALP > 900 IU/L, with serum phosphorus < 1.8 mmol/L [18]; (10) full enteral feeding time: time required for oral feeding to reach 150 mL/kg/d; (11) time to reach the standard of total calories: time required for the sum of enteral nutrition (EN) and PN calories to reach 110 kcal/kg/d; (12) GV g/kg/d [19] = (1000 × Ln [WN/W1])/(dn–d1), Wn: weight of the infant at the day of discharge (g); W1: weight of the infant after delivery at the first day (g); Dn: length of hospitalization in days (d); D1: length of regaining BW in days (d); (13) diagnostic criteria of NRDS, NEC, IVH, and PVL were referred to Practice of Neonatology (5^th^ ed.) [20].

### Statistical methods

Statistical analysis was performed using the SPSS 23.0 software (SPSS, Inc., Chicago, IL, USA). The counting data are expressed by a percentage sign (%), and the differences were detected using chi-square test or Fisher’s exact test. The distribution of the data was assessed for normality using the Kolmogorov–Smirnov test. The measurement data of abnormal distribution was shown as *M* (*Q1*, *Q3*), and their differences were analyzed using the rank-sum test. The independent samples *t*-test was used to analyze the mean comparison of two independent groups. The clinical outcomes of children were analyzed by univariate analysis, and subsequently, the risk factors related to PNAC were analyzed by unconditional multivariate logistic regression analysis. A *P*-value < 0.05 was considered significant.

## Results

### General information

**Fig. 1:**
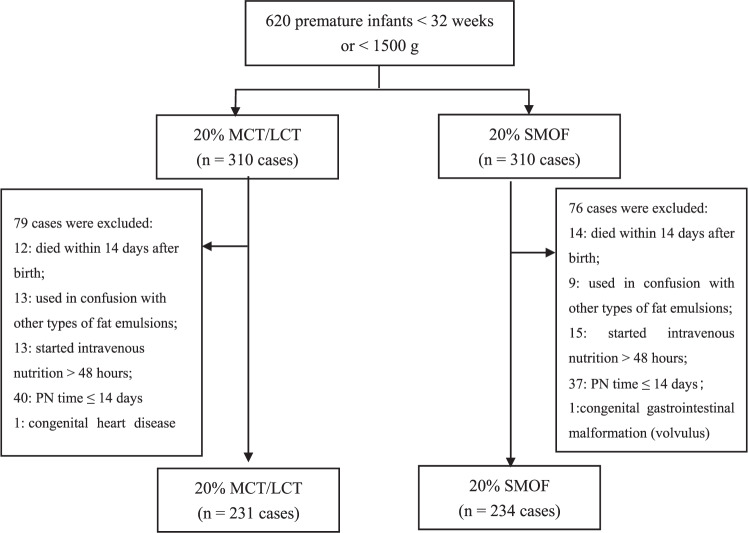
Flow chart of included and excluded patients.

During the study period, 620 preterm infants were randomly assigned to the MCT/LCT group (*n* = 310) or SMOF group (*n* = 310), and 155 unqualified cases were excluded. Finally, there were 231 cases in the MCT/LCT group and 234 cases in the SMOF group (Fig. 1). There were no significant differences in GA, BW, gender, delivery, SGA, 5 min Apgar score, premature rupture of membranes > 18 h, the use of antenatal steroids, maternal complications, NRDS, EOS, major treatment measures, the total length of hospital stay and death between the two groups (all *P* > 0.05) (Table 2).

**Table 2:** Demographic and clinical characteristics of MCT/LCT and SMOF groups.

	20% MCT/LCT *n* = 231	20% SMOF *n* = 234	*Z/T/χ*^*2*^	*P*-value
GA, [*M* (*Q1*, *Q3*)], weeks	30.1 (28.9,31.4)	29.9 (28.5,31.1)	1.300	0.254
BW, [*M* (*Q1*, *Q3*)], grams	1267.0 (1070.0,1421.3)	1221.0 (1047.5,1400.0)	1.451	0.228
Male gender, *n* (%)	122 (52.8)	123 (52.6)	0.003	0.957
Cesarean section, *n* (%)	160 (69.3)	176 (75.2)	2.053	0.152
SGA, *n* (%)	33 (14.3)	37 (15.8)	0.212	0.645
5-min Apgar score ≤ 7, *n* (%)	20 (8.7)	26 (11.1)	0.785	0.376
Premature rupture of membranes >18 h, *n* (%)	33 (14.3)	37 (15.8)	0.212	0.645
Completed antenatal steroids^a^, *n* (%)	121 (52.4)	123 (52.6)	0.002	0.968
Invasive mechanical ventilation time, [*M* (*Q1*, *Q3*)], days	0.0 (0.0,4.3)	1.0 (0.0,4.0)	0.035	0.852
Non-invasive mechanical ventilation time, [*M* (*Q1*, *Q3*)], days	21.0 (8.0,33.0)	20.0 (8.8,34.3)	0.093	0.760
Total oxygen consumption time^b^, [*M* (*Q1*, *Q3*)], days	35.5 (19.5,55.3)	35.0 (16.8,53.0)	0.570	0.450
Gestational diabetes mellitus, *n* (%)	62 (26.8)	59 (25.2)	0.160	0.689
Gestational hypertension, *n* (%)	72 (31.2)	72 (30.8)	0.009	0.926
NRDS, *n* (%)	140 (60.06)	156 (66.7)	1.846	0.174
EOS, *n* (%)	14 (6.1)	20 (8.5)	1.060	0.303
Length of hospital stay, [*M* (*Q1*, *Q3*)], days	48.0 (37.0,62.0)	50.0 (40.0,64.0)	0.755	0.385
Death, *n* (%)	5 (2.2)	4 (1.7)	0.127	0.722

^a^Intramuscular steroids cycle in two doses of 12 mg over a 24 h period.

^b^The total oxygen consumption time included invasive mechanical ventilation, non-invasive ventilator, mask, nasal catheter, and head mask.

*GA* Gestational age, *BW* Birth weight, *SGA* Small for gestational age, NRDS was neonatal respiratory distress syndrome; EOS was early-onset sepsis.

### Comparison of nutritional status of children in hospital

There were no significant differences in the starting dose and the days to reach 3.0 g/kg/d of amino acid and fat emulsion, the duration of PN, starting enteral feeding time, breastfeeding, reaching full enteral feeding time, total calorie reaching standard time, maximum weight loss, the recovery time of birth weight and GV between the two groups (all *P* > 0.05) (Table 3).

**Table 3:** In-hospital nutrition status among MCT/LCT and SMOF groups.

	20% MCT/LCT *n* = 231	20% SMOF *n* = 234	*Z/T/Χ*^*2*^	*P*-value
Starting dose of amino acids, [*M* (*Q1*, *Q3*)], g/kg	1.9 (1.3,2.0)	1.7 (1.0,2.0)	0.075	0.784
Days to reach 3.0 g/kg/d of amino acids, [*M* (*Q1*, *Q3*)], days	4.0 (3.0,5.0)	4.0 (3.0,5.0)	0.732	0.392
Starting dose of fat emulsion, [*M* (*Q1*, *Q3*)], g/kg	1.0 (1.0,1.4)	1.0 (1.0,1.2)	1.530	0.216
Days to reach 3.0 g/kg/d of fat emulsion, [*M* (*Q1*, *Q3*)], days	5.0 (4.0,7.0)	5.0 (4.0,7.0)	0.608	0.436
Duration of PN, [*M* (*Q1*, *Q3*)], days	25.0 (18.0,34.0)	26.0 (20.0,35.3)	3.154	0.076
Starting time of enteral feeding, [*M* (*Q1*, *Q3*)], hours	25.0 (18.0,42.4)	24.0 (15.0,45.0)	0.046	0.831
Breastfeeding, *n* (%)	111 (48.1)	120 (51.3)	0.485	0.486
Days to full enteral feeding, [*M* (*Q1*, *Q3*)], days	26.0 (19.0,35.3)	28.5 (20.0,38.0)	3.370	0.066
Total caloric standard days, [*M* (*Q1*, *Q3*)], days	12.0 (8.0,19.0)	11.0 (7.0,15.3)	0.174	0.676
Loss of birth weight, [*M* (*Q1*, *Q3*)], %	6.0 (3.6,8.4)	6.9 (3.6,9.8)	3.383	0.066
Days to regain BW, [*M* (*Q1*, *Q3*)], days	9.0 (7.0,11.0)	10.0 (7.0.12.0)	2.452	0.117
GV, [*M* (*Q1, Q3*)], g/kg/d	15.2 (13.1,17.5)	14.5 (13.3,16.3)	1.141	0.285

*GV* Weight growth velocity

### Comparison of blood biochemical indexes

No significant difference was observed in the levels of TB, DB, ALT, AST, ALP, TG, CHOL, Ca, and P between the two groups at baseline (PN-0) (all *P* > 0.05). After starting to use PN (PN-1), compared with the MCT/LCT group, the percentage of neonates with a TB > 5 mg/dL, a DB ≥ 2 mg/dL, a ALP > 900 IU/L and a TG > 3.4 mmol/L were lower (*P* = 0.012, 0.043, 0.033, and 0.024, respectively) in the SMOF group. The ALT, AST, CHOL, Ca, and P were not significantly different between the two groups (all *P* > 0.05) (Table 4).

**Table 4:** Blood biochemical indexes among MCT/LCT and SMOF groups.

Blood biochemical indexes		20% MCT/LCT *n* = 231	20% SMOF *n* = 234	*Z/T*/χ^2^	*P*-value
TB mg/dL [*M* (*Q1, Q3*)]	PN-0	6.41 (5.04–7.57)	6.39 (4.93–7.66)	0.112	0.737
[*n* (%)] PN-1	> 5.00	84 (36.4)	60 (25.6)	6.252	0.012
DB mg/dL [*M* (*Q1, Q3*)]	PN-0	0.47 (0.32,0.65)	0.48 (0.34,0.63)	0.284	0.594
[*n* (%)] PN-1	TB ≤ 5.00 and DB ≥ 1.00	23 (10.0)	18 (7.7)	0.741	0.389
	≥2.00	26 (11.3)	14 (6.0)	4.110	0.043
ALT IU/L [*M* (*Q1,Q3*)]	PN-0	6.0 (3.6,7.0)	6.0 (4.0,7.0)	0.400	0.527
[*n* (%)] PN-1	≥50.0	8 (3.5)	5 (2.1)	0.753	0.386
AST IU/L [*M* (*Q1, Q3*)]	PN-0	37.6 (26.2,55.0)	37.0 (25.0,53.0)	0.171	0.679
[*n* (%)] PN-1	≥40.0	41 (17.7)	45 (19.2)	0.169	0.681
ALP IU/L [*M* (*Q1,Q3*)]	PN-0	214 (164,265)	206 (158,266)	0.173	0.678
[*n* (%)] PN-1	> 900	17 (7.4)	7 (3.0)	4.531	0.033
TG mmol/L [*M* (*Q1,Q3*)]	PN-0	0.40 (0.23,0.75)	0.39 (0.22,0.70)	0.198	0.656
[*n* (%)] PN-1	<2.26	192 (83.1)	214 (91.5)	7.292	0.007
	2.26~3.4	26 (11.3)	16 (6.8)	2.761	0.097
	> 3.40	13 (5.6)	4 (1.7)	5.067	0.024
CHOL mmol/L [*M* (*Q1,Q3*)]	PN-0	2.5 (1.90,3.30)	2.5 (1.92,3.30)	0.186	0.666
[*n* (%)] PN-1	≥5.2	8 (3.5)	7 (3.0)	0.083	0.773
Ca mmol/L [*M* (*Q1,Q3*)]	PN-0	2.0 (1.9,2.3)	2.0 (1.9,2.3)	0.551	0.458
[*n* (%)] PN-1	<2.1	16 (6.9)	25 (10.7)	2.041	0.153
P mmol/L [*M* (*Q1,Q3*)]	PN-0	1.9 (1.6,2.1)	1.9 (1.6,2.4)	0.040	0.841
[*n* (%)] PN-1	<1.8	93 (40.3)	93 (39.7)	0.013	0.910

PN-0: Indexes before parenteral nutrition; PN-1: indexes after parenteral nutrition.

*TB* Total bilirubin, *DB* Direct bilirubin, *ALT* Alanine aminotransferase, *AST* Aspartate aminotransferase, *ALP* Alkaline phosphatase, *TG* Triglyceride, *CHOL* Cholesterol, *Ca* Serum calcium, *P* Serum phosphorus.

TB, DB, ALT, AST, ALP, TG, and Chol were taken as the peak values, whereas Ca and P were taken as the lowest values during the study period.

### Univariate analysis of clinical outcomes in two groups

Univariate analysis showed that the incidence of PNAC in the SMOF group was lower (*P* = 0.032). In the subgroup of < 28 weeks, the incidence of PNAC and MBDP was also lower (*P* = 0.043 and 0.029, respectively). However, the incidence of PNAC and MBDP at > 28 weeks was not significantly different between the two groups (*P* = 0.177 and 0.991, respectively). In addition, no significant difference was observed in other clinical outcomes: PDA, ROP, NEC (Bell’s stage ≥ 2), BPD, IVH grade III–IV, FI, EUGR, and LOS (all *P* > 0.05) (Table 5).

**Table 5:** Univariate analysis of recent clinical outcomes in two groups.

	*n*	20% MCT/LCT *n* = 231	*n*	20% SMOF *n* = 234	*RR* (95%CI)	*P*-value
PNAC [*n* (%)]	231	49 (21.2)	234	32 (13.7)	0.78 (0.64–0.96)	0.032
<28 w	37	17 (45.9)	38	9 (23.7)	0.62 (0.40–0.97)	0.043
≥28 w	194	32 (16.5)	196	23 (11.7)	0.83 (0.65–1.07)	0.177
PDA [*n* (%)]	231	124 (53.7)	234	141 (60.3)	1.14 (0.95–1.37)	0.152
<28 w	37	28 (75.7)	38	26 (68.4)	0.83 (0.47–1.44)	0.484
≥28 w	194	96 (49.5)	196	115 (58.7)	1.20 (0.99–1.47)	0.069
hsPDA	231	38 (16.5)	234	40 (17.1)	1.02 (0.80–1.31)	0.853
Drug or surgical treatment	231	34 (14.7)	234	39 (16.7)	1.08 (0.83–1.41)	0.564
ROP [*n* (%)]	231	112 (48.5)	234	107 (45.7)	0.95 (0.79–1.14)	0.551
<28 w	37	29 (78.4)	38	33 (86.8)	1.32 (0.79–2.18)	0.333
≥28 w	194	83 (42.8)	196	74 (37.8)	0.90 (0.74–1.10)	0.311
ROP requiring intervention	231	10 (4.3)	234	7 (3.0)	0.84 (0.56–1.26)	0.442
NEC(Bell stage ≥2) [*n* (%)]	231	12 (5.2)	234	16 (6.8)	1.17 (0.76–1.81)	0.457
<28 w	37	4 (10.8)	38	2 (5.3)	0.72 (0.39–1.33)	0.376
≥28 w	194	8 (4.1)	196	14 (7.1)	1.39 (0.79–2.44)	0.196
Surgical treatment of NEC	231	8 (3.5)	234	5 (2.1)	0.80 (0.52–1.25)	0.386
BPD [*n* (%)]	231	138 (59.7)	234	151 (64.5)	1.11 (0.92–1.33)	0.287
<28 w	37	33 (89.2)	38	36 (94.7)	1.39 (0.75–2.58)	0.376
≥28 w	194	105 (54.1)	196	115 (58.7)	1.10 (0.80–1.20)	0.365
IVH grade III– IV [*n* (%)]	231	65 (28.1)	234	64 (27.4)	0.98 (0.83-1.25)	0.849
<28 w	37	15 (40.5)	38	9 (23.7)	0.69 (0.44–1.07)	0.118
≥28 w	194	50 (25.8)	196	55 (28.1)	1.06 (0.84–1.34)	0.611
PVL [*n* (%)]	231	30 (13.0)	234	44 (18.8)	1.27 (0.95–1.70)	0.086
<28 w	37	4 (10.8)	38	7 (18.4)	1.42 (0.63–3.21)	0.352
≥28 w	194	26 (13.4)	196	37 (18.9)	1.25 (0.91–1.70)	0.142
FI [*n* (%)]	231	103 (44.6)	234	93 (39.7)	0.91 (0.75–1.09)	0.290
<28 w	37	19 (51.4)	38	22 (57.9)	1.14 (0.72–1.80)	0.569
≥28 w	194	84 (43.3)	196	71 (36.2)	0.86 (0.71–1.05)	0.153
EUGR [*n* (%)]	231	127 (55.0)	234	134 (57.3)	1.05 (0.87–1.26)	0.619
<28 w	37	21 (56.8)	38	20 (52.6)	0.92 (0.58–1.46)	0.720
≥28 w	194	106 (54.6)	196	114 (58.2)	1.07 (0.88–1.31)	0.483
LOS [*n* (%)]	231	26 (11.3)	234	33 (14.1)	1.15 (0.85–1.55)	0.356
<28 w	37	6 (16.2)	38	4 (10.5)	0.80 (0.45–1.40)	0.469
≥28 w	194	20 (10.3)	196	29 (14.8)	1.25 (0.88–1.78)	0.181
MBDP [*n* (%)]	231	13 (5.8)	234	5 (2.1)	0.68 (0.50–0.91)	0.051
<28 w	37	10 (27.0)	38	3 (7.9)	0.57 (0.38–0.85)	0.029
≥28 w	194	3 (1.5)	196	2 (1.0)	0.83 (0.11–3.97)	0.991

*PNAC* Parenteral nutrition associated cholestasis, *PDA* Patent ductus arteriosus, *hsPDA* Hemodynamically significant patent ductus arteriosus, *ROP* Retinopathy of prematurity, *NEC* Necrotizing enterocolitis, *BPD* Bronchopulmonary dysplasia, *IVH* Intraventricular hemorrhage, *PVL* Paraventricular leukomalacia, *FI* Feeding intolerance, *EUGR* Extrauterine growth retardation, *LOS* Late-onset sepsis (laboratory confirmed), *MBDP* Metabolic bone disease of prematurity.

### Multivariate logistic regression analysis of clinical outcomes in two groups

Multivariate logistic regression analysis showed that the incidence of PNAC in the SMOF group was still lower (a*RR*: 0.38, 95% CI: 0.20–0.70, *P* = 0.002), and the incidence of MBDP was also lower (a*RR*: 0.12, 95% CI: 0.19–0.81, *P* = 0.029). However, no significant difference existed in PDA, ROP, NEC (Bell’s stage ≥ 2), BPD, IVH grade III–IV, PVL, FI, EUGR, and LOS between the two groups (all *P* > 0.05) (Table 6).

**Table 6:** Multivariate logistic regression analysis of clinical outcomes in two groups.

	20% MCT/LCT *n* = 231	20% SMOF *n* = 234	a^*^RR (95%CI)	*P*-value
PNAC [*n* (%)]	49 (21.2)	32 (13.7)	0.38 (0.20–0.70)	0.002
PDA [*n* (%)]	124 (53.7)	141 (60.3)	1.43 (0.94–2.18)	0.098
ROP [*n* (%)]	112 (48.5)	107 (45.7)	0.81 (0.52–1.27)	0.362
NEC (Bell’s stage ≥ 2) [*n* (%)]	12 (5.2)	16 (6.8)	0.95 (0.35–2.55)	0.911
BPD [*n* (%)]	138 (59.7)	151 (64.5)	1.28 (0.80–2.05)	0.303
IVH grade III–IV [*n* (%)]	65 (28.1)	64 (27.4)	0.87 (0.55–1.38)	0.548
PVL [*n* (%)]	30 (13.0)	44 (18.8)	1.67 (0.94–2.98)	0.081
FI [*n* (%)]	103 (44.6)	93 (39.7)	0.73 (0.47–1.13)	0.155
EUGR [*n* (%)]	127 (55.0)	134 (57.3)	1.15 (0.61–2.16)	0.671
LOS [*n* (%)]	26 (11.3)	33 (14.1)	1.38 (0.73–2.59)	0.326
MBDP [*n* (%)]	13 (5.8)	5 (2.1)	0.12 (0.19–0.81)	0.029

^*^Adjusted for gestational age, birth weight, cesarean section, invasive mechanical ventilation time, starting dose of fat emulsion, breastfeeding, starting enteral nutrition time, duration of parenteral nutrition, age of reaching total enteral nutrition, weight growth velocity, SGA, EUGR, PDA, ROP, NEC, fi, BPD, IVH, PVL, LOS, MBD, EOS, NRDS.

## Discussion

ω-6 and ω-3 LCPUFAs are essential fatty acids and important components of the cell membrane. ω-6 LCPUFAs, represented by linoleic acid, γ-linoleic acid, and arachidonic acid (ARA), have been shown to increase the levels of pro-inflammatory cytokines and decrease oxidative stress injury [21, 22]. ω-3 LCPUFAs, such as α-linolenic acid (ALA), eicosapentaenoic acid (EPA), and docosahexaenoic acid (DHA), are commonly interconverted to anti-inflammatory mediators, which have liver-protective properties and anti-inflammatory effects [22, 23]. ω-9 monounsaturated fatty acids (MUFAs) are represented by oleic acid in olive oil; which are less susceptible to lipid peroxidation than polyunsaturated fatty acids [24]. Therefore, the American Society for Parenteral and Enteral Nutrition (ASPEN) stated that ω-6 LCPUFAs are pro-inflammatory, ω-3 LCPUFAs are anti-inflammatory, and ω-9 MUFAs are immune neutral and recommended that the optimal ratio of ω-6: ω-3 LCPUFAs in immune regulation is 1-4:1 [25]. Although the ratio of ω-6:ω-3 in MCT/LCT lipid is 7:1, the reduced content of unsaturated double bonds is attributed to the increase in MCT by 50%, which play a major role in reducing lipid peroxidation and intrahepatic cholestasis [6]. SMOF lipid is a balanced mixture of MCTs, soybean oil, olive oil, and fish oils at ratios of 30%, 30%, 25%, and 15%, respectively, and is rich in antioxidant vitamin E (200 mg/L) and α-tocopherol, with an optimum ω-6:ω-3 ratio and physiologic fatty acid composition [5]. Compared with traditional soybean oil, mixed oil emulsions, such as SMOF (containing fish oil) or MCT/LCT (without fish oil), display better short-and long-term benefits for preterm infants [26, 27]. Thus, the guidelines released by ESPGHAN/ESPEN/ESPR/CSPEN in 2018 strongly recommend that pure Soybean oil ILEs should no longer be used and composite ILEs with or without fish oil should be the first-choice treatment in preterm and term neonates with PN lasting longer than a few days [28]. However, only a few prospective comparative studies are available on MCT/LCT and SMOF lipids.

The univariate analysis revealed that compared with the MCT/LCT group, the proportion of TB＞5 mg/dL (84/231 [36.4%] vs. 60/234 [25.6%], *P* = 0.012) and DB ≥ 2 mg/dL (26/231 [11.3%] vs. 14/234 [6.0%], *P* = 0.043) were lower in the SMOF group. In children with GA of less than 28 weeks, the prevalence of PNAC was less in the MCT/LCT group (*P* = 0.043), whereas no statistically significant difference was observed between MCT/LCT with SMOF groups in children with GA > 28 weeks (*P* = 0.177). Multivariate logistic regression analysis revealed that PNAC was less prevalent in the SMOF group than that in the MCT/LCT group (a*RR*: 0.38, 95% CI: 0.20–0.70, *P* = 0.002). It is suggested that SMOF can reduce the peak level of bilirubin in VPI or VLBWI and decrease the prevalence of PNAC by about one-third, especially in extremely premature infants (EPIs) whose GA < 28 weeks. PNAC is closely related to the use of amino acids and fat emulsion, the duration of PN, and the implementation of EN [29]. No significant difference was observed between the two groups in the time of amino acids, fat emulsion starting and reaching 3.0 g/kg/d, the duration of PN in the time of starting EN, breastfeeding, and reaching the total enteral feeding (all *P* > 0.05). SMOF has been confirmed to be safe in VPI/VLBWI, with liver-protective properties that can reduce the incidence of PNAC. Kasirer et al. [30] reported that the incidence of PNAC in VLBWI in the SMOF group was lower (6% vs. 13%; *P* = 0.022), and the peak level of DB was lower (3.2 vs. 7.1 mg/dL; *P* = 0.018), which was consistent with the results of this study. SMOF can reduce the incidence of PNAC, which is primarily attributed to the fact that SMOF is a mixed oil, with an optimum ω-6:ω-3 ratio (2.5:1), which is the closest to that present in breast milk (2:1). In addition, ω-3 LCPUFAs have anti-inflammatory properties and liver-protective effects; olive oil is rich in oleic acid, belongs to ω-9 MUFAs, which are less susceptible to lipid peroxidation than polyunsaturated fatty acids [24]. Additionally, SMOF contains less phytosterol and higher doses of vitamin E and α-tocopherol, which have anti-inflammatory and antioxidation properties.

Preterm infants are at a higher risk for hypertriglyceridemia than term neonates due to the lack of adequate muscle and fat reserves, which accounts for a reduced hydrolytic capacity of the lipoprotein lipase enzyme [31]. We found that the proportion of preterm infants with TG < 2.26 mmol/L in the SMOF group was higher (192/231 [83.1%] vs. 214/234 [91.5%], *P* = 0.007) and TG > 3.4 mmol/L was lower (13/231 [5.6%] vs. 4/234 [1.7%] *P* = 0.024). Pereira-da-Silva et al. [32] reported that the level of TG was significantly higher in the MCT/LCT group than in the SMOF group (181 mg/dL vs. 134 mg/dL, *P* = 0.006), which is consistent with our findings. This can be explained by the levels of ω-3 LCPUFAs in SMOF, which have been shown to accelerate the clearance of chylomicrons, thereby effectively reducing the concentration of serum triglycerides [33] and increasing triglyceride clearance and reducing adipogenesis by inhibiting the steroid response element binding protein-1 [34]. We further identified that SMOF improved lipid tolerance of VPI and VLBWI. Additionally, the dose of amino acids and lipid for PN recommend by Guidelines for Clinical Application of Neonatal Nutritional Support in China (2013) [7], is widely used in Chinese NICUs, which is more conservative than the dose recommend by the ESPGHAN/ESPEN/ESPR/CSPEN guidelines [28]. Thus, the applicability of our results may be limited in some regions where ESPGHAN/ESPEN/ESPR/CSPEN guidelines are prevalent.

MBDP is a kind of systemic bone disease caused by the deficiency of calcium, phosphorus, and matrix Gla protein (MGP). It is also known as the disorder of bone mentalism in premature infants, characterized by the decreased bone mineral content and incomplete mineralization of osteoid [35]. It is one of the common complications in premature infants; the incidence of MBDP increases with younger GA and lower BW. The prevalence of MBDP is as high as 32% and 54% in VLBWI and extremely low birth weight infants (ELBWI), respectively [36]. In this study, ALP > 900 IU/L and P < 1.8 mmol/L were used to diagnose MBDP, with a sensitivity of 100% and a specificity of 71% [18]. The results showed that the proportion of infants with ALP > 900 IU/L was higher in the MCT/LCT group (17/231 [7.4%] vs. 7/234 [3.0%], *P* = 0.033). Univariate analysis showed that the incidence of MBDP had an increasing trend (*RR*: 0.68, 95% CI: 0.50–0.91, *P* = 0.051), and in the subgroups with GA < 28 weeks, the incidence of MBDP in the SMOF group was significantly lower than that in the MCT/LCT group (*RR*: 0.57, 95% CI: 0.38–0.58, *P* = 0.029), whereas in multivariate logistic regression analysis revealed that the incidence of MBDP in the SMOF group was lower (a*RR*: 0.12, 95% CI: 0.19–0.81, *P* = 0.029), suggesting that SMOF reduced the incidence of MBDP by about two-third, and benefitted more in EPI. This is a novel finding being reported in this study. Skourolakou et al. [37] reported that the ALP level of the SMOF group was lower and P level was higher than that of soybean oil group at discharge; however, Tomsits et al. [31] and D’Ascenzo et al. [38] reported that the use of SMOF fat emulsion did not improve the level of ALP and P. Both studies used PN for a short time, only 7 to 18 days, and none of the above studies compared the incidence of MBDP between the two groups. Considering that PN duration is a risk factor for MBDP [39], different PN may lead to inconsistent results. In this study, the effect of PN duration was assessed because the median durations of PN in the two groups were 25 days and 26 days, respectively (*P* = 0.076), which was basically the same. It has been reported that ω-3 and ω-6 LCPUFAs have opposing effects on osteoclastofenesis. The ω-6 LCPUFAs can enhance osteocalcin(OC) activity by down-regulating the ratio of osteoprotegerin to receptor activator for nuclear-factor κβ ligand (*RANKL*) gene expression in osteoblasts [40]. In addition, it promotes bone marrow-derived pluripotent human mesenchymal stem cells to differentiate into adipocytes rather than osteoblasts [40]. On the contrary, ω-3 LCPUFAs, such as DHA, inhibit osteoclasts by downregulating the expression of the *RANKL* gene and also the differentiation of bone marrow mesenchymal stem cells to osteoblasts rather than adipocytes [41]. Furthermore, ω-3 LCPUFAs may reduce the production of pro-inflammatory cytokines, increase the release of insulin-like growth factor-1 (IGF-1), and improve calcium accretion in bone [41, 42]. In addition, PNAC increases the risk of MBDP [39]. Cholestasis can lead to the decrease of cholate secretion, resulting in malabsorption of fat and fat-soluble vitamins A, D, E, K [43]. Vitamin D plays an important role in regulating the metabolism of calcium and phosphorus and ensuring bone health [44]. Both OC and MGP are vitamin K-dependent proteins; they require the participation of vitamin K to conduct γ-carboxylation and bioactivity, thus, increasing bone mineralization [45]. Simultaneously, vitamins D and K have a synergistic effect on promoting bone mineralization [44]. Therefore, the deficiency of fat-soluble vitamins D and K caused by PNAC may lead to poor bone mineralization, resulting in an increased risk of MBDP. However, the exact mechanism is still unclear and needs further study on the relationship between lipid emulsion and bone metabolism.

It remains controversial whether SMOF is superior to other complications of premature infants. Persistent oxidative stress and inflammatory are common key factors leading to BPD, ROP, LOS and other complications of premature infants. SMOF contains an optimal ratio of ω-6/ω-3 LCPUFAs (2.5:1); in addition, it is rich in α-tocopherol, which has anti-inflammatory, antioxidation, and immune-regulatory effects. Animal models showed that ω-3 LCPUFAs in fish oil could reduce intestinal inflammation and improve the systemic immune response by regulating the intestinal flora [46, 47]. Thus, we speculate that SMOF may reduce the prevalence of BPD, ROP, LOS, and NEC. A randomized, double-blind, controlled study by Hsiao et al. [48] reported that the level of IL-1β and IL-6 in the serum and bronchoalveolar lavage fluid in the intervention group (SMOF) were significantly lower than those in the control group (MCT/LCT) (*P* < 0.05), and the prevalence of BPD was significantly lower in the SMOF group (13.3% vs. 36.7%, *OR* = 0.36, 95% CI: 0.21–0.86, *P* = 0.04). However, Torgalkar et al. [27]and Repa et al.[49] did not find that the application of SMOF reduced the prevalence of BPD in ELBWI and VLBWI. A small sample study by Lin et al. [50] showed no significant difference in the incidence of BPD, ROP, PDA, LOS, IVH, PVL, EUGR, and NEC between MCT/LCT and SMOF groups in ELBWI. Yang et al. [51]compared SMOF with MCT/LCT, and the incidence of PDA, sepsis, IVH, and NEC was similar in the two groups. Certain studies have reported that early complications of premature infants have certain effects on PNAC and MBDP [10, 29, 35]. In this study, no significant difference was observed in the incidence of early complications such as BPD, ROP, PDA, NEC, IVH, PVL, EUGR, FI, and LOS between the two groups. It was further verified that SMOF reduced the risk of PNAC and MBDP. These inconsistent results may be related to the sample size, subjects, the dosage of fat emulsion, the duration of PN, the nursing level of NICU, and the interaction of other diseases. The current evidence is insufficient to determine whether SMOF has advantages in reducing BPD, ROP, and other complications of premature infants.

According to the literature review, this study is the largest prospective multicenter randomized controlled study comparing MCT/LCT and SMOF, with large sample size and reliable data. Since the five centers are distributed in 5 different provinces of China, their treatment populations may be reprensentative. It provides reliable evidence for optimizing the application of PN in premature infants. However, the study has certain limitations. Firstly, the study lacked a double-blind design because the consideration is that the use of intravenous fat emulsion should be adjusted according to the blood index of neonates to ensure medical safety. Secondly, the analysis of MBDP did not consider the dose and administration mode of Ca, P, and vitamin D supplementation between the two groups. In addition, MBDP was a secondary outcome and the sample size was not calculated to evaluate differences in incidence of MBDP, which needs further research to confirm. Thirdly, the PN protocol used in this study does not follow the ESPGHAN/ESPEN/ESPR/CSPEN 2018 guidelines, which may limit the generalizability of the study’s findings. Previous studies have demonstrated that SMOF can improve the long-term neurocognitive function of preterm infants [27, 52]. Follow-up will be conducted to further confirm the benefits of using SMOF.

## Conclusions

This prospective, multicenter, randomized controlled study demonstrated that the administration of SMOF in VPI and VLBWI could reduce the risk of TB > 5 mg/dL, DB ≥ 2 mg/dL, ALP > 900 IU/L, and TG > 3.4 mmol/L during hospitalization. Thus, SMOF has better lipid tolerance, can reduce the prevalence of PNAC and MBDP, and exerts more benefits in EPI.

## Data Availability

The datasets generated during and/or analyzed during the current study are available from the corresponding author on reasonable request.
